# Insights into coastal microbial antibiotic resistome through a meta-transcriptomic approach in Yucatan

**DOI:** 10.3389/fmicb.2022.972267

**Published:** 2022-10-17

**Authors:** Francisco Guillén-Chable, Luis Alejandro Avila Castro, Zuemy Rodríguez-Escamilla, Mario Alberto Martínez-Núñez

**Affiliations:** ^1^Unidad Multidisciplinaria de Docencia e Investigación (UMDI)-Sisal, Facultad de Ciencias, Universidad Nacional Autónoma de México (UNAM), Sisal, Yucatán, Mexico; ^2^Escuela Nacional de Estudios Superiores-Mérida, Universidad Nacional Autónoma de México (UNAM), Ucú, Yucatán, Mexico; ^3^Instituto de Investigaciones en Matemáticas Aplicadas y en Sistemas (IIMAS), Universidad Nacional Autónoma de México (UNAM), Mérida, Yucatán, Mexico

**Keywords:** antibiotic resistome, metatranscriptomics, Yucatan coast, antibiotic resistance genes, bioinformatics

## Abstract

Antibiotic resistance (AR) is one of the greatest human and clinical challenges associated with different pathogenic organisms. However, in recent years it has also become an environmental problem due to the widespread use of antibiotics in humans and livestock activities. The ability to resist antibiotics comes from antibiotic resistance genes (ARGs) and our understanding of their presence in coastal environments is still limited. Therefore, the objective of the present study was to explore the presence and possible differences in the microbial resistome of four sites from the Yucatan coast through the evaluation of the composition and abundance of ARGs using a high-throughput analysis of metatranscriptomic sequences. In total, 3,498 ARGs were uncovered, which participate in the resistance to tetracycline, macrolide, rifamycin, fluoroquinolone, phenicol, aminoglycoside, cephalosporin, and other antibiotics. The molecular mechanisms of these ARGs were mainly efflux pump, antibiotic target alteration and antibiotic target replacement. In the same way, ARGs were detected in the samples but showing dissimilar enrichment levels. With respect to the sampling sites, the ARGs were present in all the samples collected, either from preserved or contaminated areas. Importantly, sediments of the preserved area of Dzilam presented the second highest level of ARGs detected, probably as a consequence of the antibiotics dragged to the coast by submarine groundwater discharge. In general, the resistance to a single antibiotic was greater than multiresistance, both at the level of gene and organisms; and multiresistance in organisms is acquired mainly by recruiting different monoresistance genes. To our knowledge, this is the first study that describes and compares the resistome of different samples of the Yucatan coast. This study contributes to generating information about the current state of antibiotic resistance on the Yucatan coasts for a better understanding of ARGs dissemination and could facilitate the management of ARGs pollution in the environment.

## Introduction

Coastal and estuarine are complex and dynamic ecosystems, and are among highly productive and biologically diverse on Earth. Within the coasts and estuaries there are ecosystem processes such as the transfer of carbon from land to sea, releasing a considerable amount of CO_2_ to the atmosphere, and sequestering carbon in sediments (Baker et al., 2015). In addition to providing more than US$14 trillion in ecosystem goods (for example, food and raw materials) per year (Rajeev et al., 2021), these ecosystems also act as terminal basins for inland emerging contaminants (ECs) (Bhattacharyya et al., 2019; Gomes et al., 2020), where besides conventional pollutants such as pesticides, polyaromatic hydrocarbons (PAHs) and heavy metals (Rajeev et al., 2021), antibiotics and antibiotic resistance genes (ARGs) are becoming more prevalent in the coastal environments (Zhu et al., 2017; Griffin et al., 2019; Imchen and Kumavath, 2020).

Initially, the problem of antibiotic resistance (AR) was confined to hospitals and human pathogens, primarily threatening human health (Weingarten et al., 2018). But substantial anthropogenic residues of antibiotics due to widespread human and livestock use result in serious environmental problems (Shuai et al., 2022). AR in bacteria is possible through many mechanisms such as impermeable barriers, modification of target proteins, efflux pumps (e.g., AcrAB-TolC), new metabolic pathways, inactivation of the antibiotic, chromosomal mutations, and changes in membrane permeability to antibiotics (Martínez-Núñez et al., 2019; Jian et al., 2021). The molecular basis of AR are ARGs that are acquired through horizontal gene transfer (HGT) mediated by mobile genetic elements (MGEs) such as plasmids (conjugation), bacteriophages (transduction), or natural transformation by extracellular DNA (Lerminiaux and Cameron, 2018). The collection of ARGs in a specific bacteria or ecological niche is called the antibiotic resistome (D’Costa et al., 2006; Wang et al., 2020; de Abreu et al., 2021).

Antibiotic resistance genes-bearing bacteria are ubiquitous in natural niches, and the resistome has been reported to be an ancient mechanism in natural environments, with evidence of its presence in 30,000-year-old frozen sediments (D’Costa et al., 2006). The production of antibiotics in bacteria is at least hundreds of millions of years old, therefore bacteria have been directly or indirectly exposed to antibiotics and their derivatives during the same period (Wright and Poinar, 2012). Along with the production of antibiotics, bacteria have developed resistance mechanisms for their self-protection against bioactive molecules produced by fungi, plants and many other organisms. Such mechanisms have moved horizontally across microbial populations and have evolved independently (Julian and Dorothy, 2010). ARGs silently circulate between natural and man-made environments, and in this process Hu et al. (2016) found that animals play an important role in the spread of ARGs, most frequently being exchanged between animal and human bacteria, followed by animal and aquatic bacteria, and then between animal and terrestrial bacteria; besides that, they can be transferred bidirectionally from non-pathogenic to pathogenic bacteria (Cytryn, 2013). But, over the past several decades the misuse and overuse of antibiotics in animal husbandry and humans, is accelerating the evolution of resistance and contributing to the resistance crisis. The dissemination pathway of ARGs include sectors such as food animals, aquaculture, industrial and household antibacterial chemicals, or clinical use (Cytryn, 2013; Hu et al., 2016). Besides the urban and hospital sewage disposal locations, high prevalence of ARGs has been reported in coastal and pristine estuaries across the globe (Hu et al., 2017; Bhattacharyya et al., 2019; Griffin et al., 2019; Imchen and Kumavath, 2020). This raises the concern that coastal and estuarine environments could be a strong reservoir for ARGs and hotspots for their natural exchange.

Yucatan is a Mexican state located in the SE portion of the Gulf of Mexico, on Yucatan Peninsula, which is a limestone platform with a surface area of about 39,524.4 km^2^ and includes 365 km of coastline along the Gulf of Mexico with a strip that reaches up to 20 km inland from the coastline (Bauer-Gottwein et al., 2011). It lacks bodies of surface-water, presenting a system of groundwater that flow into the coast. The Yucatan coast has 54.4% of the mangroves in Mexico, which carry out 46% of carbon capture and storage in the country (Adame et al., 2013; Cinco-Castro and Herrera-Silveira, 2020; Herrera-Silveira et al., 2020). Sixty percent of the coastal strip is under protection through two Biosphere Reserves, Celestún and Ría Lagartos, as well as two state Natural Protected Areas, El Palmar and Bocas de Dzilam de Bravo. In addition, the four areas have been recognized by the Convention on Wetlands of International Importance, known as the Ramsar Convention. During the last decade the Yucatan coast has suffered an increase in pollution due to primary activities, industrial discharges, excessive fishing and tourism, negatively impacting ecological, socioeconomic, and health levels. This has resulted in the presence of pollutants such as PAHs, pesticides, heavy metals, personal care products and pharmaceutical products being reported in coastal and estuarine ecosystems (Herrera-Silveira et al., 2004; Metcalfe et al., 2011; Moreno-Pérez et al., 2021). Few studies have been conducted to assess the environmental presence of ARGs in the area; to our knowledge, only the work carried out by Moore et al. (2019) in which the presence of ARGs in groundwater sites (cenotes) was evaluated. Through a metagenomic approach, they found that the most common resistance mechanisms were various multi-drug resistance efflux pumps, as well as the presence of genes for resistance to diverse antibiotics. Therefore, it is necessary to evaluate the environmental presence of ARGs, which allows knowing its distribution along the Yucatan coast, for a better understanding of the processes that originate AR in microbial communities and its possible impact on human health. The objective of this work is to identify the presence of the active antibiotic resistome and its possible differences in the microbial communities that inhabit the coast of Yucatan. To our knowledge this is the first report that uses a meta-transcriptomic analysis in order to generate information of the current status of Mexican coasts on antibiotic resistance in sites with different degrees of contamination as a result of anthropogenic activities.

## Materials and methods

### Site description and sample processing

**FIGURE 1:**
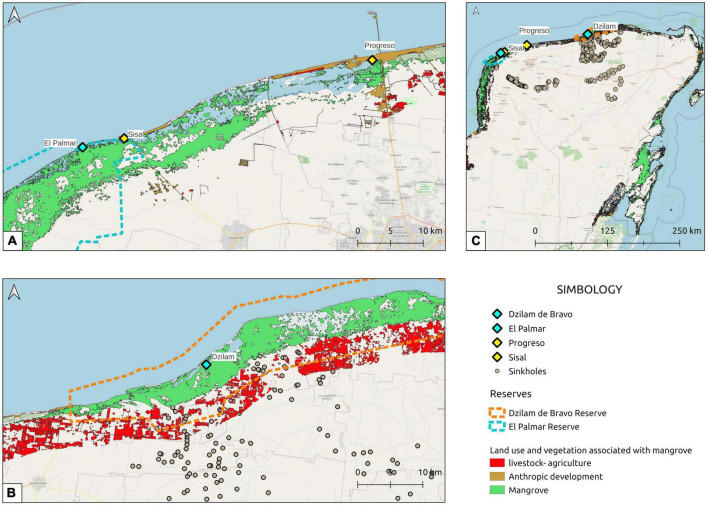
Locations of sampling sites on the Yucatan coast. **(A)** The sampling sites of El Palmar, Sisal, and Progreso on the Yucatan coast are shown. **(B)** Bocas de Dzilam sampling site and its proximity to an area of great livestock and agricultural activity are shown. **(C)** The zone of the Yucatan Peninsula and the ring of cenotes is shown, as well as the location of the four sampled sites. Yellow diamonds show contaminated sampling sites (Sisal and Progreso); while the diamonds in blue show the ecological reserves where sampling was carried out (El Palmar and Dzilam). The ring of cenotes over the Yucatan Peninsula is also shown with the gray dots.

The identification of the ARGs present in the sediments of Yucatan coast was carried out at four sites: two sites with anthropic impact, and two state ecological reserves. The selected wetlands with anthropic impact were the swamps in the towns of Sisal (21°09′43.6″ N 90°02′27.2″ W) and Progreso (21°16′37.6″ N 89°40′35.6″ W); these locations are contaminated with organic and inorganic garbage, septic tank sedimentation sludge, sanitary waste, insecticides, and petroleum hydrocarbons with the concentration of total petroleum hydrocarbons (TPH) exceeding the permissible level in coastal sediments of 70 μg/g (Suárez-Moo et al., 2020). The preserved wetlands were those located in El Palmar (21°08′56.4″ N 90°06′07.0″ W) and Bocas de Dzilam (21°27′22.2″ N 88°40′53.7″ W), which are Reserves State Ecological, in addition to Ramsar sites (Figure 1).

Three sediment samples were taken for each site, for which three points were chosen within a box meter square: one point at the center and two more at the ends. The samples were extracted approximately 20 cm deep, and 2 g of sediment were taken from each point and mixed with 6 mL of the LifeGuard Soil Preservation buffer (Qiagen, Hilde, Germany) in 50 ml sterile polypropylene tubes. After sampling, the sediments were immediately placed in a cooler with ice at 15°C and transported to the laboratory facilities to be stored refrigerated at −20°C. Sampling was carried out as detailed in the Table 1.

**TABLE 1:** Spatial and temporal details of locations sampled in this study.

Sampling site	Coordinates	Sampling replicates in 1 m^2^ surface	Date
**Anthropogenic impacted locations**			
Sisal	21°09′43.6″ N 90°02′27.2″ W	3	May, 2017
Progreso	21°16′37.6″ N 89°40′35.6″ W	3	October, 2019
**Ecological reserved locations**			
Palmar	21°08′56.4″ N 90°06′07.0″ W	3	March, 2018
Dzilam	21°27′22.2″ N 88°40′53.7″ W	3	October, 2019

### Nucleic acids extraction and transcriptomic sequencing

Nucleic acids extraction, libraries construction, and sequencing were requested from the Research and Testing Laboratory (Lubbock, TX, USA). For RNA extraction Qiagen RNeasy PowerSoil Total RNA Kit (Qiagen, Hilde, Germany) was used following the manufacturer’s recommendations. RNA sequencing (RNA seq) libraries were constructed and sequenced following a default Illumina^®^ Stranded Total RNA Prep protocol. Sequencing was done using an Illumina HiSeq 2500 platform to generate 2 × 150 bp paired-end reads. Sequencing resulted in an average yield of 58 million reads per sample.

### Meta-transcriptomics data analysis

The raw data obtained from the sequencing by RNA-seq of the triplicates for each site were first filtered to remove adapters, as well as low-quality reads (Phred score < 30), using the NGS QC Toolkit v2.3.3 software (Patel and Jain, 2012), and its program IlluQC.pl for Ilumina data using default parameters, after removing reads with poor quality 85% passed the filter. A library of Palmar samples was removed due to the low number of reads obtained, staying at the end with 11 libraries: 2 for El Palmar site, 3 for Sisal site, 3 for Progreso site, and 3 for Bocas de Dzilam site. Subsequently, the filtered reads were assembled by *de novo* assembly package Trinity (Grabherr et al., 2011). Annotation of assembled transcriptomes and taxonomic assignation was performed locally using BLASTx sequence similarity searches (Altschul et al., 1997; Altschul and Koonin, 1998) against the protein UniProtKB/Swiss-Prot database (Consortium, 2019), with a threshold of *e*-value < 10^–20^. The following command was used to carry out the annotation: blastx -query trinity_data.fasta -db Uniprot_database.fasta -out Annotations.txt -*e*value 1*e*^–20^. The RSEM v.1.3.3 package (Li and Dewey, 2011) was used to normalize the reads data to correct for library size and gene lengths bias, in order to compare count of genes among the samples from the study sites. Through RSEM we obtained the transcripts per million (TPM) values and these were used to extract ARGs counts that were unique to each site. A custom Perl script was used to extract the unique ARGs, considering as expressed only those genes with an average TPM ≥ 1.

### Antibiotic resistance genes analysis

The Comprehensive Antibiotic Resistance Database v.3.2.0 (CARD) (Alcock et al., 2020), which includes 4,970 hand-curated ARG reference sequences, 60 drugs class and 7 resistance mechanism was used to classify and quantify ARGs. To identify ARGs in our Trinity-assembled transcripts with an average TPM ≥ 1, a BLASTx was performed locally against CARD database. Command line used for this was: blastx -query trinity_annotated.fasta_-db CARD_database.fasta -out ARGs_Annotations.txt -*e*value 1*e*^–20^. Transcripts were identified as an ARG-like sequence if the best BLASTx hit had a similarity over 90%, an alignment longer than 25 amino acids and *e*-value of 1*e*^–20^. The resulting transcripts annotated as ARGs were classified into (a) 60 classes of antibiotics to which the genes are resistant, and (b) 7 mechanisms of resistance according to the CARD database. In addition, previously identified ARGs were classified into those that present resistance to one antibiotic (monoresistance) or to several antibiotics (multiresistance). Taxonomic annotations of transcriptomes previously made were also used to identify organisms with mono- or multi-resistance. Organisms identified as multiresistant were in turn classified as native multiresistant and multiresistant by recruitment. The native multiresistant organisms were those that presented genes with resistance to several antibiotics; while multiresistant organisms by recruitment are those organisms that have acquired several monoresistance genes to different antibiotics. The datasets of this study can be found in NCBI online repositories with the accession numbers to BioProject PRJNA851022.

### Statistical analysis

To carry out the statistical analysis and visualization of the data, the free software environment for statistical computing and graphics R v.4.2.1 was used (R Core Team, 2021). Principal component analysis (PCA) and graphs were done with *factoMineR* v.2.4 and *factoextra* v.1.0.7 packages, respectively. Heatmap graphs were produced using *pheatmap* v.1.0.12 package. The statistical significance of the difference in the number of ARGs between the samples was performed using the TPM value and applying the Kruskal–Wallis Rank Sum and Pairwise Wilcoxon Rank Sum Tests using the *stats* v.4.3.0 package of R. Stacked bar plot and boxplot graphs were produced with *ggpubr* v.0.4.0 and *ggplot2* v.3.3.6 packages. The *phyloseq* v1.4 package was used to calculate the richness and the Shannon index, for which both the total abundance and the diversity of the resistance genes were considered.

## Results

### Distribution and abundance of antibiotic resistance genes in conserved and contaminated sites of the coast

**FIGURE 2:**
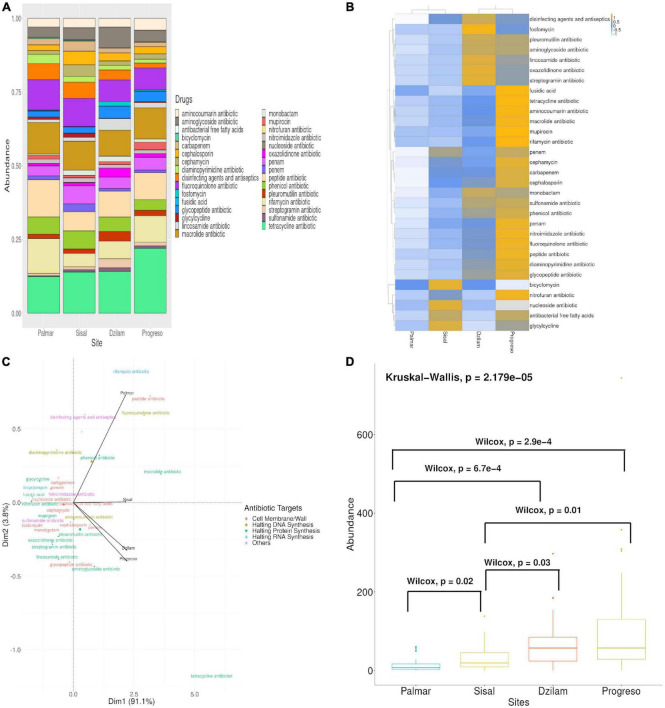
Antibiotic resistance genes (ARGs) found on the Yucatan coast. **(A)** Relative abundance of ARGs classes found in the Yucatan coast. **(B)** Heatmap representing the number of ARGs classes found in each site. **(C)** Principal component analysis (PCA) analysis represents ARGs classes compositional differences between the sites. **(D)** Boxplot, Kruskall–Wallis, and Wilcox test of ARGs abundance in the different sites.

A total of 3,498 ARGs were identified from all sediment samples, which were distributed into 31 antibiotic resistance classes represented 6,705 times (Figure 2A and Supplementary Tables 1–4); with a total of 163 (4.65%), 322 (9.20%), 1,073 (30.67%), and 1,940 (55.46%) ARGs for Palmar, Sisal, Dzilam, and Progreso, respectively. Of the 60 antibiotic resistance classes contained in the CARD database, we identified 31 (51.6%) of them in our data; and of these 31 classes, 27 are present in all sites. Only antibacterial free fatty acids and nitrofuran antibiotic were present in Sisal and Progreso sites; fusidic acid was present in Palmar, Dzilam, and Progreso; and nucleoside antibiotic was present only in Palmar. The top ten different AR classes with the highest proportion of genes were tetracycline (12.2%), peptide (9.9%), macrolide (9.22%), fluoroquinolone (9.0%), rifamycin (6.6%), phenicol (5.9%), penam (5.1%), cephalosporin (3.8%), aminoglycoside (3.6%) antibiotics and disinfecting agents and antiseptics (3%). While aminocoumarin (2%), glycopeptide (1.7%), and lincosamide (1%) antibiotics are present in a less abundant manner. The most abundant genes were outer membrane proteins such as TolC, ABC transporter ATP-binding protein, regulatory proteins such as FleR, AtoC, QseF, and NifA, among others (Supplementary Tables 1–4). We measured alpha diversity metrics within each site in terms of ARG classes and gene richness (i.e., number of classes and genes represented) and diversity (Shannon index, i.e., evenness of the abundance levels among classes and genes, summarized in Table 2 and Supplementary Figures 1A,B).

**TABLE 2:** Richness and diversity index (Shannon) of antibiotic resistance gene (ARG) classes and genes by sampling site.

	ARG classes		Genes	
Sites	Richness	Shannon	Richness	Shannon
Palmar	28	2.79	143	4.82
Sisal	30	2.95	215	5.12
Dzilam	28	2.96	462	5.74
Progreso	30	2.77	731	6.01

A comparative analysis was performed between the four samples to identify where the highest frequency of ARGs is found, and if there are similar groups with the same abundance of ARGs. The results indicate that the average level of ARGs detected in the samples of the study sites range from lower to higher proportion in the following order: Palmar, Sisal, Dzilam, and Progreso (Figure 2B). The correlation of the ARGs detected in the four samples showed the formation of two clusters among the analyzed sites. On the one hand, the samples from Progreso and the preserved site of Dzilam were grouped together, these being the sites with the highest abundance of ARGs. While the samples from the town of Sisal and the conserved site of El Palmar form a separate group, these sites are the ones with a lower abundance of ARGs (Figure 2B).

We performed a Principal Component Analysis (PCA) to assess the differences in composition between the sites. The analysis showed a clear separation between the samples due to their composition of ARGs, observing that the Progreso contaminated site is more closely related to the preserved Dzilam site, while the Sisal and El Palmar sites are separated as single points in space (Figure 2C). In order to evaluate the existence of differences in the distribution of ARGs at each site, a Kruskall–Wallis test was performed; the statistical result showed a difference (*p*-value < 2.179*e*^–5^) between the abundance levels of the samples. To carry out the identification of which groups presented a significant difference in the content of ARGs, a paired Wilcox test was performed, finding a significant difference (*p*-value < 0.05) between all the groups, except for the samples of Progreso and Dzilam (Figure 2D).

### Classes of antibiotic resistance mechanisms in the Yucatan coast

**FIGURE 3:**
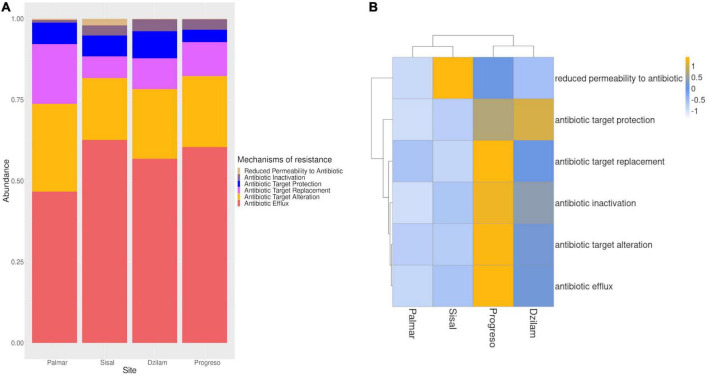
Mechanisms of resistance to antibiotics found on the Yucatan coast. **(A)** Relative abundance of mechanisms of antibiotic resistance. **(B)** Heatmap representing the number of mechanisms of resistance to antibiotics found in each site.

From our data, it was possible to identify six of the seven classes of antibiotic resistance mechanisms registered in the CARD database present in the Yucatan coast (Figure 3A). The only mechanism not identified in our analysis was resistance by absence, which is associated with the deletion of gene, usually a porin. In order to identify where the greatest frequency of antibiotic resistance mechanisms is found, as well as the existence of similarities between the sampling sites in their resistance mechanisms, we carried out a comparative analysis of the four sites. From the results obtained, it is observed that the samples from Progreso and Dzilam were grouped, which present the highest abundance of resistance mechanisms. While the samples from Sisal and the conserved site of El Palmar form a separate group, these sites are the ones with a lower abundance of resistance mechanisms (Figure 3B). At the Sisal site, reduced antibiotic permeability mechanism had the highest frequency of occurrence, higher than at the other sites; this is a mechanism that is usually carried out through reduced porin production that can provide resistance. The grouping patterns observed in our analysis of resistance mechanisms is consistent with what was observed in the analysis of ARGs, in which the sites of Progreso and Dzilam are the ones with the highest abundance of both, grouping together. While the Palmar and Sisal sites were grouped separately, presenting the lowest abundance of ARGs and antibiotic resistance mechanisms.

### Monoresistance and multiresistance to antibiotics in the Yucatan coast

**FIGURE 4:**
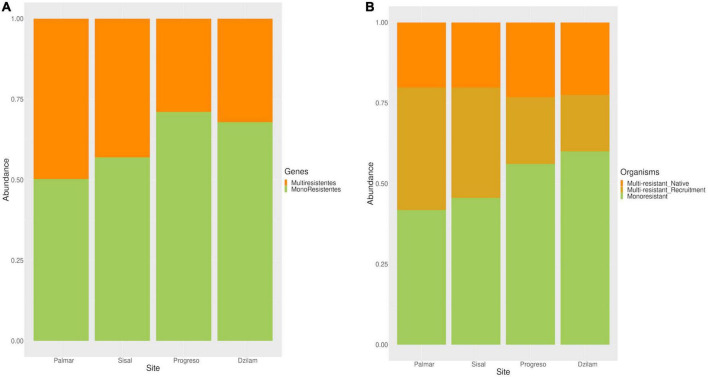
Antibiotic resistance genes (ARGs) and organisms with monoresistant and multiresistant to antibiotics found on the Yucatan coast. **(A)** Relative abundance of ARGs monoresistant and multiresistant found in each site. **(B)** Relative abundance of organisms with monoresistant and multiresistant; multi-resistance in organisms was classified as native and recruited.

In order to be able to differentiate the types of resistance to antibiotics that are present in the sites sampled on the Yucatan coast, we carried out the classification of ARGs and organisms into monoresistant and multiresistant. Monoresistance, both in organisms and in genes, refers to the ability to resist only one type of antibiotic. Regarding the multiresistance, in the case of the organisms it was divided into two: (a) the native multiresistance and (b) the recruited one. Native multiresistance is that conferred by genes with the ability to resist two or more types of antibiotics; while recruited multiresistance is that which is obtained by acquiring two or more genes with monoresistance capability. On the other hand, multiresistant genes are those that confers the ability to resist two or more types of antibiotics. From our data, the genes with monoresistance ability are the most abundant, with ranges from 50 to 70% in the following order: El Palmar, Sisal, Dzilam, and Progreso. While the values for the abundance of multiresistant genes are the opposite, being Progreso the site with the lowest abundance, and El Palmar the site with the highest abundance of multiresistant genes (Figure 4A). Regarding the abundance of monoresistant and multiresistant organisms, the trend seems to reverse. There is a greater number of multiresistant organisms in Palmar, followed by Sisal, then Progreso, and finally Dzilam (Figure 4B). The composition of multiresistance in organisms is in turn divided into two; the sites of Progreso and Dzilam present a slightly greater abundance of native multiresistant than that observed in El Palmar and Sisal, while the last two sites have the same level of abundance (Figure 4B). The abundance of multiresistant organisms by recruitment is higher at El Palmar and Sisal, and lower at the Progreso and Dzilam sites. In general terms, at the gene level, the resistance mechanism to a single antibiotic is the one that predominates in all the sampling sites, both in the contaminated sites and in the conserved ones. At the level of organisms, what is observed is that in the sampling sites of El Palmar and Sisal, the organisms that present multiresistance are the ones that predominate, while in Progreso and Dzilam the monoresistant organisms predominate.

## Discussion

### Antibiotic resistance genes dissemination in Yucatan coast

Wide use of antibiotics, as well as the residues they generate, exert selective pressure on microorganisms, thus resulting in ARGs enrichment, both at the organismal and environmental levels (Wang et al., 2020). This problem has gone beyond hospitals or urban environments ceasing to be only a human health problem but also begin to become an environmental health problem, with which the ARGs can spread in both directions. The present study aimed to explore the distribution of ARGs in coast ecosystems, and to our knowledge, this is the first report of antibiotic resistomes carried out in the Yucatan Peninsula using data from transcriptional high-throughput sequencing from bacterial communities.

Most studies on estuarine and coastal ARGs have been conducted in Europe and East Asia (Zheng et al., 2021); and the main ARGs reported in these studies were sulfonamides, fluoroquinolones, tetracyclines, macrolides, chloramphenicols, and β-lactams (Imchen et al., 2018; She et al., 2018; Imchen and Kumavath, 2020; Zhou et al., 2022). The only study carried out in the Yucatan Peninsula area on antibiotic resistance was conducted by Moore et al. (2019) using metagenomic sequencing data. In that study, antibiotic resistance genes against fluoroquinolones, β-lactams, macrolides, N-glycosides, glycopeptides, aminoglycosides are reported. The results from our metatranscriptomic analysis of the resistome showed that the same classes of ARGs are present in greater abundance in all sampled sites. In addition we find cephalosporin, rifamycin, peptide antibiotic, diaminopyrimidine antibiotic, aminocoumarin as the most abundant (Supplementary Tables 1–4). The identification of 31 ARG classes shows that a wide variety of them are found in coastal wetlands, not only in those sites with direct contamination by human activities, but also in pristine areas. This agrees with what was reported by Imchen and Kumavath (2020) for the pristine and human-disturbed mangrove datasets from China, India, and Malaysia where 27 ARG classes were found regardless of environmental status.

From the diversity analysis, we observed that in terms of the ARG classes, the sites with the greatest richness were Sisal and Progreso, which are sites with direct anthropogenic impact because they are urban sites. Regarding the uniformity of the ARG classes abundance levels, the Sisal and Dzilam sites were the ones that had the highest index of diversity of Shannon (Table 2 and Supplementary Figure 1). It is noteworthy that although the Progreso site had one of the highest richness of ARG classes, its level of diversity is low due to the dominance of some classes such as tetracycline, macrolides, peptides, and rimfamycin. What would be indicating an intensive use of these antibiotics among the population that lives in Progreso. When we perform the diversity analysis of the genes associated with antibiotic resistance, what is observed is that the highest richness and the highest Shannon diversity index are found in the Progreso and Dzilam sites. From Figure 1 we can observe that these sites are the most impacted by human activities; in the case of Progreso, it is the urban site with the highest population density, while Dzilam is the site with the greatest impact of livestock activities in the eastern part of the state. Exposure to a greater variety of antibiotics generates a greater diversity of genes associated with their resistance. Contaminated sites have been reported to have the highest alpha diversity of ARGs, such an increase in diversities shows that human intervention enriches a large pool of them. ARGs enrichments in human-intervened mangroves could lead to accelerated ARGs dissemination among microbes of different phylogeny through horizontal gene transfer (Laroche et al., 2009; Imchen and Kumavath, 2020). The most abundant genes were outer membrane proteins such as TolC, which is a major outer-membrane antibiotic efflux channel in Gram-negative bacteria (Housden et al., 2021); and regulatory proteins that form part of two-component system such as FleR, which is part of FleS–FleR system and has a role in intrinsic antibiotic resistance (Gooderham and Hancock, 2009), or AtoC involved in resistance to aminoglycoside antibiotics through AtoC-AtoS system (Matta et al., 2007; Supplementary Tables 1–4). In the case of El Palmar, the lower exposure to antibiotics results in lower gene richness and lower gene diversity (Table 2 and Supplementary Figure 1), which would seem to indicate that in pristine sites the diversity of ARGs is low. In summary, these results indicate that the coastal wetlands of Yucatan constitute a large reservoir of ARGs, from the west of the Peninsula to the east, where pristine and contaminated areas are represented.

### Abundance of emerging contaminants in pristine areas

The abundance of ARGs along the Yucatan coast is not homogeneous. From the comparative analysis carried out between the four sampled sites it was possible to identify that the highest abundance of ARGs is found in the Progreso and Dzilam sites, both in the same heatmap cluster and without significant differences in terms of the abundance of ARGs detected in them (Figures 2B,D). While the Palmar and Sisal sites were clustered separately, presenting a significantly lower abundance of ARGs compared to the Progreso and Dzilam sites (Figures 2B,D). When we evaluate the differences of the sampled sites in terms of the composition of the ARGs classes present in them through a PCA, we observe that the anthropogenic impacted site of Progreso is more closely related to the conserved site of Dzilam (Figure 2C). The greater abundance of ARGs in the Progreso site is explained by the impact of human activities resulting from the urbanization of this place and by its greater population density, much higher than that of the Sisal settlement (Figure 1A). It is noteworthy that the second site with the highest abundance of ARGs is the conserved site of Dzilam. Among the most abundant ARGs found in Dzilam, these belong to classes of antibiotics used in livestock activities, such as fluoroquinolones, macrolides, and tetracyclines (Kim and Carlson, 2007; Kim et al., 2017; Van et al., 2020; Palacios et al., 2021) which shows that their use in such activities generates a problem that affects coastal areas. This problem is aggravated, since Mexico is the ninth consumer country of veterinary antimicrobials and access to them without a prescription is easy (Tiseo et al., 2015; Zaidi et al., 2015). In the specific case of pig farms in southeastern Mexico, severe and fatal multidrug-resistant, extended-spectrum cephalosporin-resistant *Salmonella typhimurium* infections in infants have been traced to swine and contaminated pork meat (Zaidi et al., 2007.). From our data, it is observed that the ARG resistant to cephalosporins was one of the most abundant in Dzilam.

When carrying out the analysis of the possible sources of contamination of Dzilam, we observe in the map of Figure 1B (red rectangles) that the distribution of livestock activities in the state of Yucatan is carried out with greater intensity in the eastern part of the state, just behind the Dzilam Conservation Area. Extensive animal breeding may be related to the spread of antibiotics over large areas, since 90% of antibiotics administered to farm animals are excreted in the form of the original compound or metabolites through feces or urine, this being one of the ways in which antibiotics are released to the environment (Palacios et al., 2021). Therefore, a possible cause of the presence of ARGs in Dzilam is the submarine groundwater discharge (SGD) that crosses the livestock production area and could be dragging the antibiotic residues that would reach the Dzilam coast, affecting the ecological reserve (Figure 1B). Karst aquifers, such as the one in the Yucatan Peninsula, have been documented as having a high capacity to store and transport contaminants, such as untreated sewage from leaking septic systems, urban runoff, and agricultural fertilizers, from sources to areas of potential exposure such as are the estuaries; and Dzilam is an important area of SGD on the north coast of Yucatan (Murgulet et al., 2020). The consequence of this problem is the contamination of the sites declared as environmental reserves, such is the case of Bocas de Dzilam, which was the second site that presented the highest abundance of ARGs, as well as high Shannon diversity indices for the ARG classes and genes (Figure 2D, Table 2, and Supplementary Figure 1). It has been reported that low concentrations of ARGs can be found in pristine sites, or significantly lower than in environments directly impacted by urban/agricultural activity (Pruden et al., 2006). And this is what we observed in the conserved site of El Palmar, which presented the lowest abundance of ARGs. The presence of ARGs in Palmar may be the product of the diffuse flow of the SGD that reaches the coast through the Celestún estuary (Murgulet et al., 2020). Although it has also been reported that the presence of ARGs in the sediments of pristine mangroves could be attributed to the multiple functions that antibiotics perform in microbial communities at a concentration lower than the inhibitory concentration, such as in competition between species, transcriptional modulation of virulent, metabolic and adaptive functions, or SOS response (Sengupta et al., 2013). Although it has been reported that some ARG abundances are often positively correlated with antibiotic concentrations in the environment (Luo et al., 2010; McKinney et al., 2010), the identification and quantification of the antibiotics present in the Yucatan coast is necessary to complement the results presented here from a transcriptomic perspective.

### Efflux pumps as the main antibiotic resistance mechanisms

Next, we ask what mechanisms are involved in antibiotic resistance in the localities sampled and, also, if there is any variation. We found 6 antibiotic resistance mechanisms, and the main mechanism that occurred in all sites was the antibiotic efflux, around a 50% (Figure 3A), which is accomplished through efflux pumps located at the plasmatic membrane. This is in line with what was reported by Imchen and Kumavath (2020) on mangrove sediments, in which efflux pumps covered over one-third of the resistome and were significantly enriched in the human intervened mangrove sediments, as we observed in samples from the contaminated sites of Progreso and Sisal (Figures 3A,B). Efflux pumps are known to confer some level of resistance to all types of antibiotics by striking a balance between reducing the intracellular concentration of the antibiotic to prolong survival time, which in turn creates a window for the potential development of resistance to antibiotics through spontaneous mutations (Morar and Wright, 2010; Machado et al., 2018; Imchen and Kumavath, 2020). A significant fraction of ARGs in the analyzed sediments also acts by the mechanisms of antibiotic target alterations and antibiotic target replacement, which are the second and third most abundant mechanisms; which is consistent with that reported in other studies (Griffin et al., 2019; Wang et al., 2020; Jian et al., 2021; Zheng et al., 2021). Antibiotic target alteration is carried out through mutations or enzymatic modifications of the antibiotic target, and antibiotic target replacement is achieved with a replacement or substitution of the target of action of the antibiotic. Among the less abundant mechanisms of antibiotic resistance are reduced permeability and inactivation of antibiotics, the latter being mainly associated with resistance to common anthropogenic antibiotics, such as aminoglycosides, β-lactams, and macrolides (Chen et al., 2013). From the comparative analysis carried out between the four sites, we can observe that in Progreso the least represented resistance mechanism is the reduction of permeability, while the other five mechanisms have a high representation. In the case of Dzilam, the target protection mechanism was the one that presented the greatest representation. Finally, we can observe that in Sisal the only mechanism with the greatest representation was permeability reduction, as well as we can observe that it was the only site in which the six resistance mechanisms identified in our study were present. While in El Palmar none of the mechanisms had an overrepresentation over the others (Figure 3B) indicating that in pristine sites the abundance of both ARGs and antibiotic resistance mechanisms is lower.

### Monoresistance and multiresistance in coastal environments

Classifying ARGs into those that confer resistance to one antibiotic (mono) and those that confer resistance to several antibiotics (multi), we observe that at least 50% of the genes are monoresistant in all the sites sampled. Reported data considering the contribution of monoresistance genes versus multiresistance genes in antibiotic resistance in coastal environments is limited. Monoresistance genes have been described in individual genomes and their mechanism of resistance to a single drug class may be due the mutation of target proteins; for example, DNA gyrases and topoisomerases IV genes in *Escherichia coli* confers resistance against quinolones (Moon et al., 2010). Other mechanisms include the enzymatic inactivation of the drug such as enzymatic phosphorylation, acetylation, adenylation, or enzymatic hydrolysis (Smith and Baker, 2002; Egorov et al., 2018). Finally, another intriguing mechanism is the bypassing of the target as in resistance to vancomycin in enterococci, where the substrate to which the antibiotic binds is replaced by an ester structure (Nikaido, 2009). From our data we observed that the highest abundance of monoresistant genes is in the contaminated sampling sites such as Sisal and Progreso (Figure 4A). The exception is the conserved Dzilam site, in which the abundance of monoresistant genes is even higher than that observed in samples from the Sisal-contaminated site. What was observed is possibly a consequence of contamination processes with antibiotic residues that reach the coast of Dzilam through submarine discharges, as mentioned before. In contrast, the main mechanism of multidrug resistance has been associated with active transporters that pumps out from the cell a broad spectrum of chemically different substances, i.e., efflux pumps (Higgins, 2007; Nikaido, 2009). When we compare the abundance of monoresistant against multiresistant organisms, we observe a small increase in the proportion of the latter. And again, the multiresistant organisms were more abundant in the sites with the lowest abundance of ARGs, those are El Palmar and Sisal. Multiresistance in bacteria can arise due a single biological mechanism conferring resistance to multiple drugs, or because several genes that confer resistance to several antibiotics are genetically linked on the chromosome or a plasmid (Nikaido, 2009; Jacopin et al., 2020). As detailed in the Section “Results,” our data show that the multiresistance observed in the organisms identified in the analyzed sites is constituted by recruitment of monoresistant genes in at least half of them. An observation derived from our data is that in places with lower abundance of ARGs, the presence of multiresistant organisms is greater, as in the samples from El Palmar and Sisal (Figure 2D and Figure 4A). Why are multiresistant organisms found in greater abundance in these sites? Several hypotheses have been proposed for the proliferation of multiresistance, including that it evolves more easily when it is less costly than having a combination of individual resistances, and when a bacterial population is exposed to a variety of antibiotics (Chang et al., 2015; Jacopin et al., 2020). More studies are necessary to understand the processes of mono and multiresistance to antibiotics in coastal systems, since so far, the studies carried out have been done in clinical or epidemiological models.

## Conclusion

Our study suggests that antibiotic resistance genes are widely dispersed in our sampling sites on the Yucatan coasts. And in addition, there are some sampling places considered as conserved natural areas that present a great abundance of ARGs. The presence of ARGs on the Yucatan coast can be explained by the submarine groundwater discharge that characterize the Yucatan Peninsula (i.e., the ring of cenotes) which acts as a large drag force for pollutants from distant places. The indiscriminate use of antibiotics in clinical environments and in livestock activities in the region generates residues that are dragged to the coastal environments of the Yucatan Peninsula. Said residues are deposited in the sediments of the mangroves, which are serving as active sinks for ARGs, turning them into hot spots of antibiotic resistance in the region. The use of NGS tools in Mexico has been little explored for the monitoring of emerging pollutants in coastal areas, such as ARGs. This is the first study carried out on the coast of Yucatan with these tools, which allows laying the foundations for subsequent environmental monitoring studies. The identification and quantification of the antibiotics present in the Yucatan coast is necessary to complement the results presented here from a transcriptomic perspective.

## Data availability statement

The data presented in this study are deposited in the NCBI-BioProject repository, accession number PRJNA851022.

## Author contributions

MAM-N and ZR-E: conceptualization. MAM-N: methodology, resources, supervision, project administration, and funding acquisition. LAAV and MAM-N: data collection. FG-C and MAM-N: formal analysis. FG-C, ZR-E, and MAM-N: manuscript writing. All authors contributed to the article and approved the submitted version.

## Supplementary material

Supplementary Figure 1 Plots of Shannon diversity for antibiotic resistance gene (ARG) classes and genes.

Supplementary Table 1 This table containing sequences annotated as antibiotic resistance genes (ARGs) in Palmar sample site, containing CARD IDs, ARO IDs, antibiotic classes, and mechanisms of action per line.

Supplementary Table 2 This table containing sequences annotated as antibiotic resistance genes (ARGs) in Sisal sample site, containing CARD IDs, ARO IDs, antibiotic classes, and mechanisms of action per line.

Supplementary Table 3 This table containing sequences annotated as antibiotic resistance genes (ARGs) in Dzilam sample site, containing CARD IDs, ARO IDs, antibiotic classes, and mechanisms of action per line.

Supplementary Table 4 This table containing sequences annotated as antibiotic resistance genes (ARGs) in Progreso sample site, containing CARD IDs, ARO IDs, antibiotic classes, and mechanisms of action per line.
