# Supplementary material for: Insights into coastal microbial antibiotic resistome through a meta-transcriptomic approach in Yucatan
Source: Front Microbiol. 2022 Oct 17;13:972267. doi: 10.3389/fmicb.2022.972267 (PMC9618888; doi:10.3389/fmicb.2022.972267)
Supplement: Supplementary Figure 1 — Plots of Shannon diversity for antibiotic resistance gene (ARG) classes and genes. [file Data_Sheet_1.PDF]

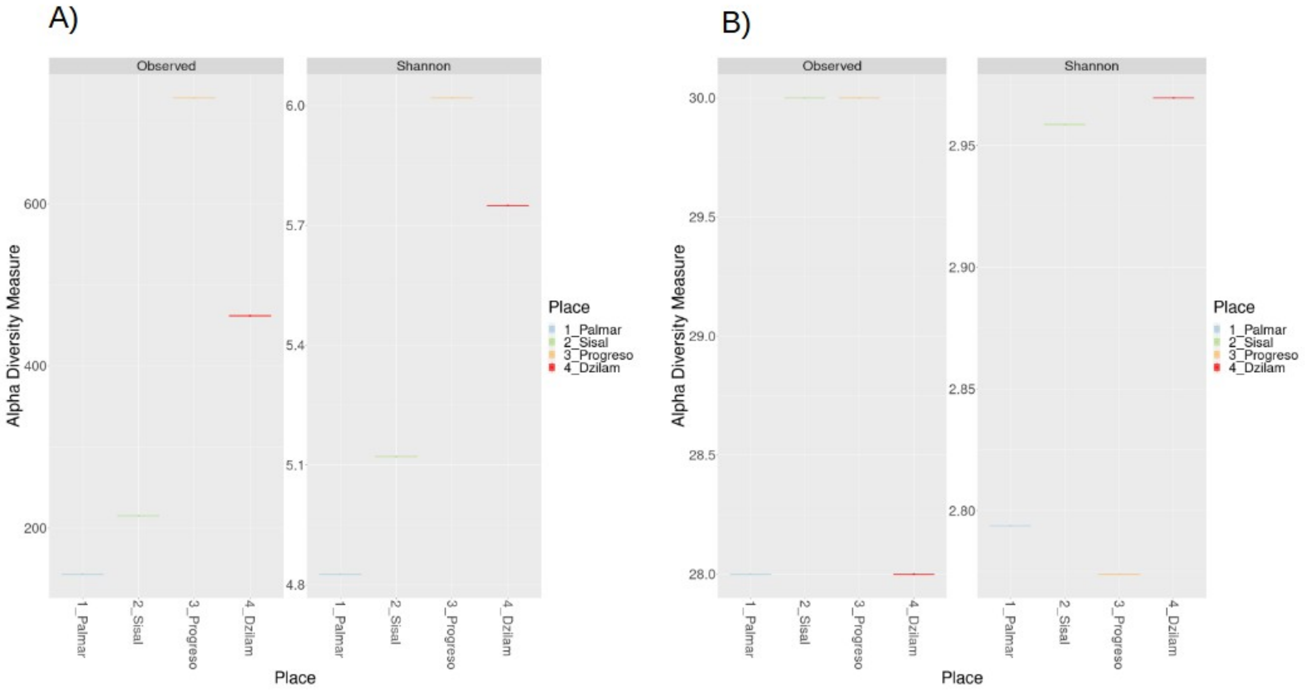

Supplementary figure 1. Calculated Shannon diversity detailed on plots A) for ARG classes and in B) for genes. Calculation was made as described on Materials and methods.
